# Assessing the limitations of paraformaldehyde fixation for accurate cell surface receptor measurement

**DOI:** 10.3389/fphar.2026.1727410

**Published:** 2026-03-24

**Authors:** Emma Bernard, Amandine Wahart, Nicolas Pataluch, Céline Galés, Véronique Pons

**Affiliations:** INSERM, UMR 1297, Institut des Maladies Métaboliques et Cardiovasculaires (I2MC), Université de Toulouse, Toulouse, France

**Keywords:** cell surface quantification, ELISA, G protein-coupled receptor, paraformaldehyde fixation, permeabilization

## Abstract

**Introduction:**

Quantifying cell surface receptors, distinct from the total cellular receptor pool, which includes both intracellular and membrane-bound compartments, is imperative in cell biology and pharmacology. Cell surface receptors are pivotal for initiating signaling cascades in response to extracellular stimuli, making their accurate quantification critical for understanding initial cellular responses and signal transduction mechanisms.

**Methods:**

Whole-cell Enzyme-Linked Immunosorbent Assays (ELISA) are commonly used to assess receptor expression in both permeabilized and non-permeabilized cells. Using angiotensin II type 1 receptor (AT1-R) and sorting nexin 12 (SNX12) as models for cell surface and intracellular proteins, respectively, we evaluated the impact of paraformaldehyde (PFA) fixation on protein quantification.

**Results:**

We found that a fixation step with 4% paraformaldehyde (PFA), widely employed across biological protocols, significantly permeabilizes cellular membranes. This results in the unintended detection of intracellular proteins in non-permeabilized cells, thereby leading to inaccurate measurements of plasma membrane receptor expression. Reducing the PFA concentration to 1% substantially improved the accuracy of cell surface receptor quantification by limiting membrane permeabilization while maintaining total receptor content. This adjustment was particularly important when assessing the surface expression of certain GPCR mutants that are retained intracellularly. However, the effect was protein-dependent, as 1% PFA caused inadequate protein immobilization and partial loss of some cytosolic proteins.

**Discussion:**

These findings underscore the necessity for tailored fixation protocols, depending on the protein of interest, in order to preserve cell surface integrity and ensure reliable quantification of proteins in diverse biological studies.

## Introduction

Cell surface receptors are involved in a wide array of biological processes. Among them, G protein-coupled receptors (GPCRs) represent the largest superfamily of cell surface transmembrane receptors and are one of the most successful targets for drug discovery. These receptors translate extracellular stimuli into intracellular signals to orchestrate downstream physiological responses and dysregulation of their signaling is implicated in a multitude of human pathologies, ranging from cardiovascular diseases to cancer (Liu et al., 2024). Since these receptors achieve most of their functions by primarily binding their ligands at the plasma membrane, receptor activity is directly related to their expression at the cell surface. Hence, the accurate quantification of receptor expression exclusively at the cell surface, i.e., on intact cells, while avoiding the detection of intracellular receptors, is of critical importance for the pharmacological characterization of these receptors. This approach provides valuable insights into receptor function and ligand-receptor interactions but also into receptor trafficking and regulation, as well as drug targeting, and disease mechanisms. For instance, many genetic disorders are associated with mutations that cause intracellular retention and impaired trafficking of receptors to the plasma membrane (Morello et al., 2000; Lubrano-Berthelier et al., 2003). Consequently, evaluating cell surface receptor expression is an essential step in studying the pharmacological properties of mutant receptors.

Historically, in the field of GPCR research, radioligand binding assays have been the first reliable and sensitive approach for quantifying receptor expression in GPCR studies (Lefkowitz et al., 1970). However, these assays exhibit specific limitations, primarily due to their indirect assessment of receptor expression, which relies solely on the ability of a radioactive ligand to recognize the receptor. Moreover, the emergence of biased ligands in recent years (Ma et al., 2025) has raised concerns about the capacity of radioligand binding to accurately quantify the entire receptor population at the cell surface. An additional drawback of this method is linked to the use of radioactive materials, which are subject to increasingly stringent safety regulations aimed at reducing or eliminating their use. Over the last years, alternative strategies have emerged and whole-cell Enzyme-Linked Immunosorbent Assay (ELISA) appeared as an easy and widely used, powerful alternative method to monitor cell surface expression of GPCRs (Pandey et al., 2019). ELISA was typically performed on cells stably or transiently transfected to express GPCRs bearing extracellular N-terminal tags, such as HA, Myc or FLAG tags. This approach utilized the high specificity and sensitivity of anti-tag antibodies, enabling the comparative analysis of the expression levels of two different receptors fused to the same tag.

Basically, the ELISA protocol starts with an initial step of chemical fixation, commonly achieved through paraformaldehyde (PFA) treatment on the living GPCR-expressing cells. In cell biology and immunohistochemistry techniques, fixative reagents play a critical role in immobilizing cellular structures and molecules for study and are used to preserve the morphology, structure, and biochemical integrity of cells and tissues. Among them, PFA is a commonly used fixative, usually preferred for fixation because it rapidly penetrates cells, while preserving cellular morphology and structures. The PFA fixation process involves crosslinking, which refers to the formation of covalent bonds between proteins, DNA, and other cellular components, stabilizing the cell architecture. This fixation method effectively immobilizes and conserves target molecules of interest. When used alone, PFA fixation maintains the integrity of cell-surface structures and does not inherently permeabilize cells, thus hindering access to intracellular targets. To study intracellular components, such as proteins or enzymes, it becomes necessary to permeabilize the cell membrane, allowing antibodies or other detection reagents to access intracellular compartments. However, PFA fixation can compromise cell membrane integrity, depending on its concentration and the duration of exposure, potentially increasing membrane permeability. In cell biology, a 4% PFA concentration is conventionally used for fixation, primarily based on its effectiveness in preserving cellular morphology. This concentration is widely employed in GPCR studies requiring cell fixation, including those focused on GPCR drug screening. Nevertheless, the suitability of 4% PFA has not been systematically evaluated for specific assessment of cell surface receptor expression.

In this study, we evaluated the effect of 4% PFA fixation on the specific detection of GPCR expression at the cell surface of HEK293T cells using ELISA method. Our findings demonstrate that performing cell fixation using the classically used 4% PFA triggered unwanted membrane permeabilization leading to inappropriate detection of intracellular proteins. To improve cell surface quantification of GPCR, we reduced the fixative concentration to 1% PFA, which significantly improved the detection accuracy of cell surface transmembrane receptors. However, when tested on several intracellular proteins in permeabilized cells, fixation with 1% PFA resulted in a decreased assessment of total expression of some–though not all–cytoplasmic proteins compared to fixation with 4% PFA. This highlights insufficient immobilization of certain intracellular components at the lower PFA concentration and a concomitant membrane permeabilization leading to a loss of soluble proteins during extensive washing steps. Consequently, these results further emphasize the need for optimized fixation protocols tailored to specific experimental objectives, balancing the surface receptor preservation with the integrity of intracellular proteins. Overall, these results underscore the importance of fine-tuning fixation conditions to achieve optimal cellular preservation and accurate protein quantification in biological studies.

## Results

### Classic fixation using 4% paraformaldehyde triggers cell membrane permeabilization

**FIGURE 1:**
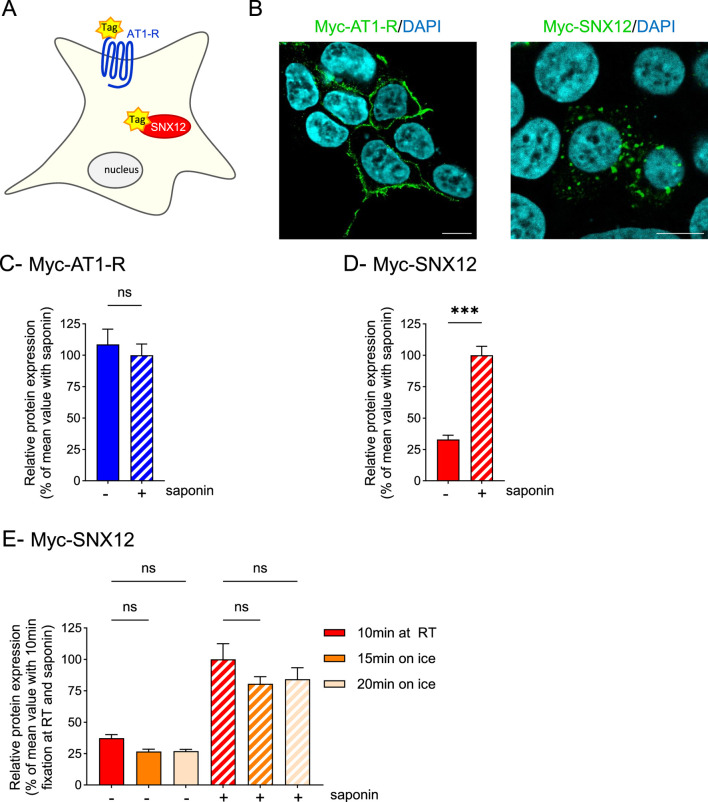
Paraformaldehyde fixation triggers membrane permeabilization and detection of intracellular proteins **(A,B)**. HEK293T cells were transfected using a lipid-based transfection reagent with vectors encoding Myc-tagged AT1-R or SNX12 **(A)** and then processed for immunofluorescence **(B)**. Scale bar: 10 μm. **(C,D)**. HEK293T cells were transfected using a lipid-based transfection reagent with vectors encoding Myc-AT1-R **(C)** or Myc-SNX12 **(D)**, then fixed using 4% PFA, permeabilized (+) or not (−) with saponin and protein expression was quantified by whole-cell ELISA. Data represent the mean ± s.e.m. of four independent experiments. Statistical significance was assessed using an unpaired t-test (***p < 0.001; ns, not significant). **(E)**. HEK293T cells were transfected using a lipid-based transfection reagent with vectors encoding Myc-SNX12 and fixed using 4% PFA for 10 min at room temperature (RT) or for 15 or 20 min on ice. Then, cells were permeabilized (+) or not (−) with saponin and protein expression was quantified by whole-cell ELISA. Data represent the mean ± s.e.m. of four independent experiments. Statistical significance was assessed using Brown-Forsythe and Welch ANOVA followed by Dunnett’s post-tests (ns, not significant).

We performed the classically described ELISA protocol to quantitatively assess the expression of GPCRs at the cell surface on non-permeabilized cells. We used the angiotensin II type 1 receptor (AT1-R) as a control GPCR known to be prominently expressed at the plasma membrane. Additionally, we used sorting nexin 12 (SNX12), a soluble cytoplasmic protein associated with the endosomal compartment, as a negative control due to its exclusive intracellular localization and lack of expression on the cell surface. Both AT1-R and SNX12 were N-terminally fused with Myc tag and transiently expressed in HEK293T cells (Figure 1A). We first validated the cellular expression and localization of these two proteins by immunofluorescence on permeabilized cells using an anti-Myc antibody (Figure 1B). As expected, Myc-AT1-R was mainly detected at the cell surface, demonstrating efficient trafficking of the receptor to the plasma membrane whereas Myc-SNX12 was exclusively confined to intracellular vesicles, in agreement with its specific localization in early endosomes (Pons et al., 2012).

We next quantified protein expression specifically at the plasma membrane using the well-established ELISA protocol on non-permeabilized cells. For this purpose, HEK293T cells were transiently transfected with plasmids encoding either Myc-AT1-R or Myc-SNX12. Cells were fixed with 4% PFA for 10 min at room temperature and detection was carried out using the same anti-Myc antibody for both proteins. Cell surface *versus* total expression of Myc-tagged proteins was then assessed. To evaluate total protein levels, a permeabilization step was subsequently performed using saponin, a detergent that removes lipids from membranes allowing penetration of the antibodies and thus intracellular detection of proteins. As shown in Figure 1C, the expression of Myc-AT1-R assessed in the presence of saponin (hatched blue bar) was similar to that obtained in the absence of saponin (solid blue bar), indicating efficient trafficking of the receptor to the plasma membrane. In contrast, Myc-SNX12 expression showed a substantial detection in the absence of saponin, corresponding to 32.89% ± 3.39 of total expression (Figure 1D). These results suggest that fixation with 4% PFA induced significant permeabilization of the plasma membrane, rendering it unsuitable for analysis on non-permeabilized cells. Considering that the fixation step can also be performed on ice to better preserve cellular structures (Pandey et al., 2019), we performed similar experiments but fixing the cells with 4% PFA for 15 or 20 min on ice, instead of the standard 10-min fixation at room temperature. Even with ice-based fixation, we still observed plasma membrane permeabilization, with 26.64% ± 1.96% and 26.92% ± 1.55 of total Myc-SNX12 signal detected in non-permeabilized cells after 15 or 20 min on ice, respectively–values comparable to those obtained at room temperature. Consequently, ice-based fixation did not reduce membrane permeabilization, as we still detected an unexpected proportion of intracellular Myc-SNX12 (Figure 1E).

To confirm that the unexpected intracellular protein detection using 4% PFA was not dependent on a specific tag and/or primary antibody used, we fused both AT1-R and SNX12 to the HA tag instead of the Myc tag and performed identical experiments to those depicted in Figure 1. As expected, HA-AT1-R and HA-SNX12 exhibited comparable localization to their Myc-tagged counterparts when using the anti-HA primary antibody (Supplementary Figure S1A). Consistent with our observations for Myc-tagged proteins, ELISA-based assessment of HA-AT1-R expression still showed comparable expression levels in both non-permeabilized and permeabilized cells (Supplementary Figure S1B), while HA-SNX12 consistently exhibited robust detection in non-permeabilized cells (Supplementary Figure S1C), indicating that membrane permeabilization with 4% PFA occurred regardless of the specific tag or the antibody used for protein detection.

To exclude any potential influence of the lipid-based transfection reagent–X-tremeGENE^TM^ 9 DNA transfection reagent–on cellular membrane permeability, we conducted parallel experiments using an alternative transfection reagent, namely linear polyethylenimine (PEI), a synthetic cationic polymer. Consistent with the results obtained with the lipid-based transfection reagent (Figures 1C,D), Myc-AT1-R was similarly detected whether the cells were permeabilized or not (Supplementary Figure S2A) while Myc-SNX12 still exhibited significant detection in non-permeabilized cells, accounting for 24.78% ± 4.31 of total Myc-SNX12 expression (Supplementary Figure S2B). The extent of cell membrane permeabilization and subsequent intracellular protein detection appeared to be comparable in cells transfected with the synthetic cationic polymer PEI (Supplementary Figure S2B) or with the lipid-based transfection reagent (Figure 1D). These results indicated that the nature of the transfection reagent has a negligible effect on the degree of PFA-induced membrane permeabilization.

Furthermore, since the composition of the plasma membrane is highly dependent on the physiological function of the cell and varies among tissues (Pradas et al., 2018; Hoejholt et al., 2019), we next investigated the impact of 4% PFA fixation on a different cell type. To address this, we examined whether 4% PFA fixation resulted in similar permeabilization in HeLa cells, which are epithelial human cells derived from a cervical carcinoma and widely used cell line in biology. In confocal imaging, in contrast to its major localization at the plasma membrane in HEK293T cells, Myc-AT1-R exhibited an additional intracellular distribution pattern upon expression in HeLa cells (Supplementary Figure S3A), reflecting its trafficking along the secretory pathway and suggesting different efficiencies of cell surface trafficking between the two cell lines. In agreement, only half of the total AT1-R expression was detected at the cell surface in non-permeabilized cells by the ELISA method, compared to permeabilized cells (Supplementary Figure S3B), confirming that receptor trafficking to the plasma membrane is less efficient in HeLa cells compared to HEK293T cells. Consistent with our findings in HEK293T cells, intracellular Myc-SNX12 was still detected in HeLa cells after fixation with 4% PFA, in the absence of saponin (Supplementary Figure S3C), providing further evidence of PFA-induced membrane permeabilization. Remarkably, this detection in non-permeabilized cells accounted for 25.73% ± 5.59 of the total protein expression. In this specific case, where the GPCR displays partial intracellular localization, the accurate assessment of receptor levels at the cell surface can be significantly compromised by membrane permeabilization induced by 4% PFA fixation. These results demonstrated that the 4% PFA fixation step induces cell membrane permeabilization irrespective of the cell type.

### Decreasing paraformaldehyde concentration reduces cell membrane permeabilization but results in the loss of cytoplasmic content

**FIGURE 2:**
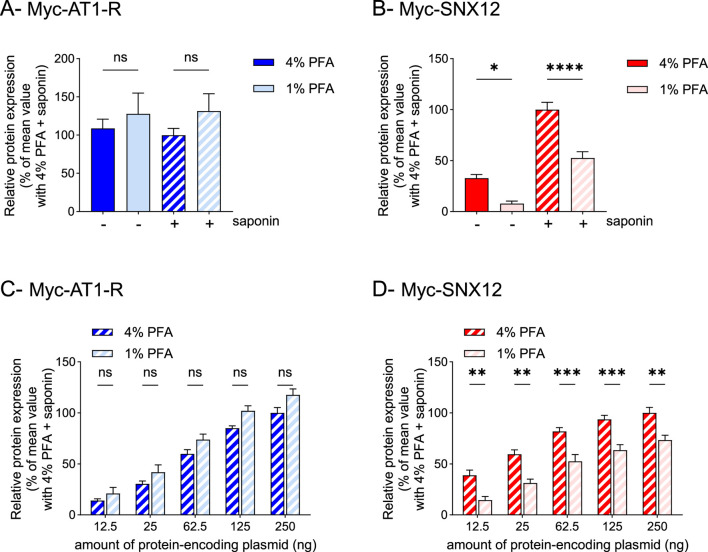
Decreasing paraformaldehyde concentration reduces membrane permeabilization but is associated with loss of cellular content **(A,B)**. HEK293T cells were transfected using a lipid-based transfection reagent with vectors encoding Myc-AT1-R **(A)** or Myc-SNX12 **(B)**. Then, cells were fixed using 4% PFA (data from Figures 1C,D respectively) or 1% PFA, permeabilized (+) or not (−) with saponin and protein expression was quantified by whole-cell ELISA. Data represent the mean ± s.e.m. of four independent experiments. Statistical significance was assessed by one-way ANOVA followed by Sidak’s post-tests (*p < 0.05; ****p < 0.0001; ns, not significant). **(C,D)**. HEK293T cells were transfected using a lipid-based transfection reagent with increasing amounts of vectors encoding Myc-AT1-R **(C)** or Myc-SNX12 **(D)** (ranging from 12.5 to 250 ng/well). Then, cells were fixed using 4% PFA or 1% PFA, permeabilized with saponin and protein expression was quantified by whole-cell ELISA. Data represent the mean ± s.e.m. of four independent experiments. Statistical significance was assessed using two-way ANOVA followed by Sidak’s post-tests (**p < 0.01; ***p < 0.001; ns, not significant).

To improve the ELISA detection method and reduce intracellular protein detection, we lowered the PFA concentration to 1% during fixation. In HEK293T cells expressing Myc-AT1-R, the detection of the receptor in non-permeabilized cells was similar, whether the cells were fixed with 4% or 1% PFA (Figure 2A). By contrast, in cells expressing Myc-SNX12, the detection of the intracellular protein in non-permeabilized cells–in the absence of saponin–was significantly reduced in cells fixed with 1% PFA compared to cells fixed with 4% PFA (Figure 2B).

However, although this encouraging result might, at a first glance, suggest a decrease in cell membrane permeability, it turned out that the total expression of Myc-SNX12 protein, measured in permeabilized cells and expected to remain constant regardless of fixation conditions, was also significantly reduced in cells fixed with 1% PFA (Figure 2B), leading to an erroneous evaluation of Myc-SNX12 levels. This reduction is unlikely to result from cell loss due to insufficient fixation, as Myc-AT1-R levels remained stable under both fixation conditions (Figure 2A). We therefore hypothesized that the inaccurate assessment of total Myc-SNX12 expression was primarily due to a loss of cellular content during the ELISA experimental procedure. Indeed, the paraformaldehyde fixation step is known to immobilize proteins by achieving chemical crosslinks between free amino groups, thereby forming a matrix that traps proteins and lipids (Kiernan, 2000). Insufficient crosslinking at 1% PFA likely impaired retention of intracellular components. Thus, the intracellular cytoplasmic protein SNX12, which is a peripheral membrane-associated protein, was insufficiently retained and consequently lost during successive washing steps along with cytoplasmic content. Conversely, the transmembrane protein AT1-R that was tightly embedded in the lipid bilayer through its seven transmembrane domains was sufficiently immobilized and thus consistently detected in saponin-treated cells regardless of the fixative concentration used.

This hypothesis was further supported by results showing the total expression of the two proteins of interest in permeabilized cells fixed with 4 or 1% PFA after transfecting increasing amounts of plasmids encoding these proteins (Figures 2C,D). Notably, whatever the expression level of Myc-AT1-R, the overall expression of the receptor remained consistent irrespective of whether the cells were fixed with 4% or 1% PFA (Figure 2C). In contrast, the total expression of Myc-SNX12 was consistently significantly reduced at each expression level, in cells fixed with 1% PFA compared to those fixed with 4% PFA (Figure 2D). This observation underscores a critical underestimation of total Myc-SNX12 protein content in cells fixed with 1% PFA, which cannot be attributed to cell loss but rather to a loss of cellular content.

To enhance protein immobilization during the 1% PFA fixation step, we extended the fixation time period and performed similar experiments but fixed the Myc-SNX12 expressing cells during 15 or 20 min instead of 10 min. Our results indicated that prolonged fixation with 1% PFA resulted in higher detection of total Myc-SNX12 expression, although it remained incomplete, with levels still remaining significantly lower compared to fixation with 4% PFA (Supplementary Figure S4, hatched bars). Under these conditions, the quantification of intracellular protein expression in non-permeabilized cells indicated persistent membrane permeabilization (Supplementary Figure S4, solid bars).

Overall, these results underscore the challenge of optimizing fixation conditions to achieve both efficient cell fixation and the absence of membrane permeabilization when specifically assessing the cell surface expression of a protein of interest.

Using an alternative tag (Supplementary Figure S5A), a different transfection reagent (Supplementary Figure S5B) or a distinct cell line (Supplementary Figure S5C) still led to inaccurate assessment of total SNX12 expression, likely due to a significant loss of intracellular content since SNX12 detection was consistently lower in saponin-permeabilized cells fixed with 1% than with 4% PFA.

**FIGURE 3:**
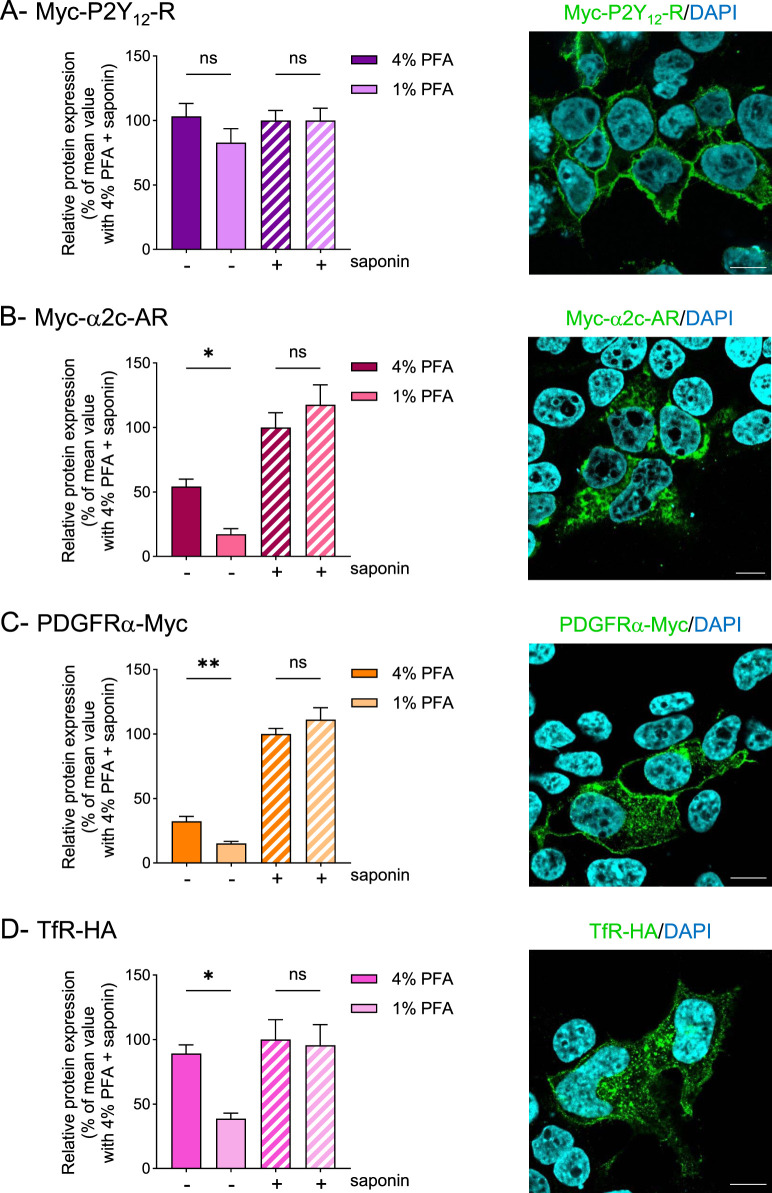
Decreasing paraformaldehyde concentration decreases membrane permeabilization while preserving transmembrane receptor detection HEK293T cells were transfected using a lipid-based transfection reagent with vectors encoding Myc-P2Y_12_-R **(A)**, Myc-α2c-AR **(B)**, PDGFRα-Myc **(C)** or TfR-HA **(D)**. Then, cells were fixed using 4% or 1% PFA, permeabilized (+) or not (−) with saponin and protein expression was quantified by whole-cell ELISA. Data represent the mean ± s.e.m. of six **(A,C)** or five **(B,D)** independent experiments. Statistical significance was assessed using one-way ANOVA followed by Sidak’s post-tests or Brown-Forsythe ANOVA followed by Dunnett’s post-tests (*p < 0.05; **p < 0.01; ns, not significant). For each transfection, protein localization was assessed by immunofluorescence (right panels). Scale bar: 10 μm.

**FIGURE 4:**
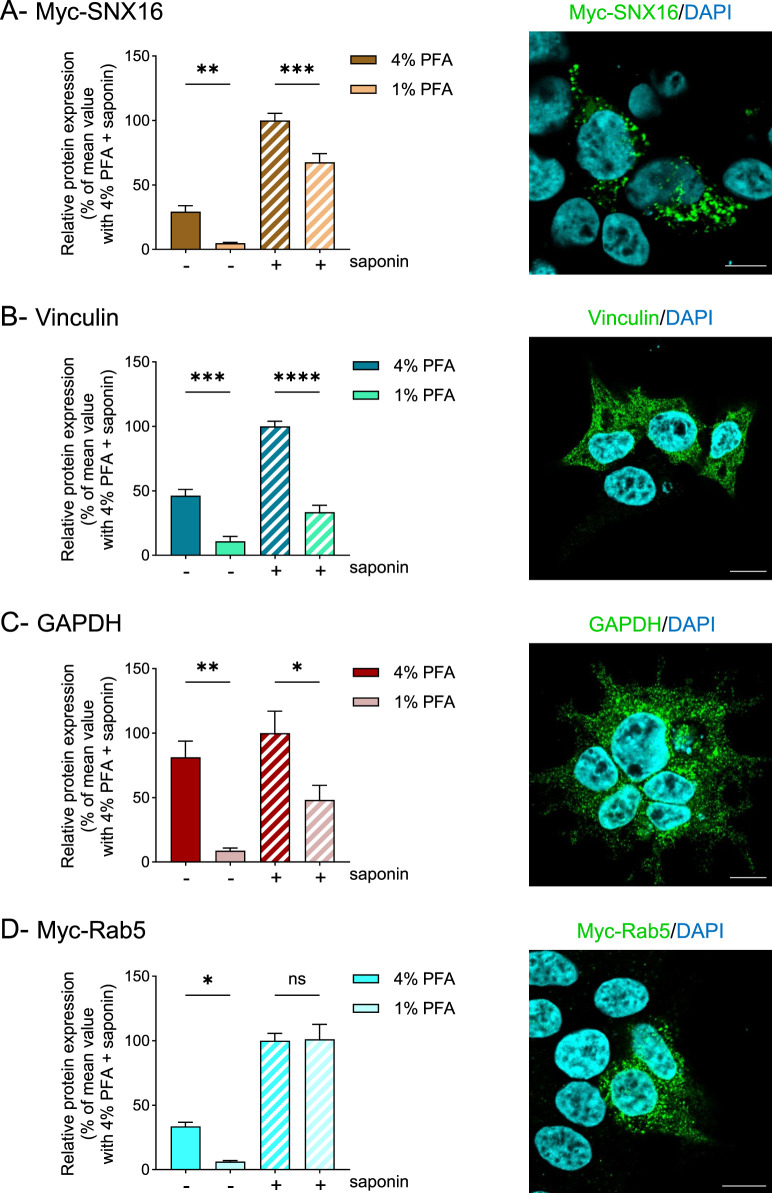
The effect of reduced paraformaldehyde concentration on total intracellular protein detection varies according to protein type. HEK293T cells were transfected using a lipid-based transfection reagent with vectors encoding Myc-SNX16 **(A)**, Myc-Rab5 **(D)** or left untransfected **(B,C)**. Then, cells were fixed using 4% or 1% PFA, permeabilized (+) or not (−) with saponin and protein expression was quantified by whole-cell ELISA. Data represent the mean ± s.e.m. of five **(A,D)**, or four **(B,C)** independent experiments. Statistical significance was assessed using one-way ANOVA followed by Sidak’s post-tests (*p < 0.05; **p < 0.01; ***p < 0.001; ****p < 0.0001; ns, not significant). For each transfection, protein localization was assessed by immunofluorescence (right panels). Scale bar: 10 μm.

To generalize these findings, we extended our investigation to additional transmembrane receptors (Figure 3) and intracellular cytoplasmic proteins (Figure 4) under the same experimental conditions. We first assessed two additional GPCRs: the purinergic receptor P2Y_12_ (Myc-P2Y_12_-R) and the α2c-adrenergic receptor (Myc-α2c-AR). Like AT1-R, Myc-P2Y_12_-R predominantly localized to the plasma membrane in HEK293T cells (Figure 3A, right panel) and varying PFA concentrations did not affect receptor detection by ELISA, regardless of saponin presence (Figure 3A). In contrast, Myc-α2c-AR exhibited substantial intracellular localization, likely within the secretory pathway, indicating less efficient targeting to the plasma membrane (Figure 3B, right panel), as known for this receptor (Filipeanu et al., 2011). In this specific case, where the receptor shows partial intracellular localization, the specific quantification of its cell surface levels can be significantly compromised by membrane permeabilization induced by 4% PFA fixation. In agreement, we found that lowering the PFA concentration decreased receptor detection in non-permeabilized cells, without altering the assessment of total receptor expression in the presence of saponin (Figure 3B).

We then evaluated additional transmembrane proteins beyond GPCRs, including single-pass membrane receptors such as PDGF receptor α (PDGFRα) and transferrin receptor (TfR). As a control, PDGFRα was fused with a Myc tag at its intracellular C-terminal region, which should prevent surface detection. Contrary to this expectation, fixation with 4% PFA produced a detectable signal in non-permeabilized cells, accounting for 32.3% ± 3.87 of the total protein expression, confirming membrane permeabilization. Reducing the PFA concentration to 1% improved the accuracy of surface receptor assessment by markedly decreasing the surface signal–although it remained detectable–while preserving total receptor detection in saponin-permeabilized cells (Figure 3C).

TfR exhibited both surface and cytoplasmic localization, with strong intracellular signal due to constitutive cycling between the plasma membrane and endosomal compartments (Figure 3D, right panel). However, when assessing TfR fused to an HA tag at its extracellular C-terminal region (TfR-HA), the receptor was prominently detected at the plasma membrane in non-permeabilized cells after 4% PFA fixation (Figure 3D), which is inconsistent with its predominantly intracellular localization at steady state. Similar to Myc-α2c-AR, lowering the PFA concentration significantly improved the assessment of surface TfR by reducing plasma membrane detection without affecting total expression in permeabilized cells, more accurately reflecting the receptor distribution observed in immunofluorescence.

Next, we investigated additional intracellular cytoplasmic proteins, including SNX16, another member of the sorting nexin family, which, like SNX12, primarily localizes to endosomal membranes *via* a phosphoinositide-binding domain (Brankatschk et al., 2011). Similar to SNX12, but unlike multispanning membrane receptors, the total expression of Myc-tagged SNX16 was inaccurately quantified and underestimated in cells fixed with 1% PFA compared to 4% PFA (Figure 4A). We also analyzed other intracellular proteins, including the endogenous cytoplasmic/cytoskeletal-associated protein vinculin (Figure 4B) and the cytoplasmic enzyme GAPDH (Figure 4C). In both cases, fixation with 1% PFA led to a significant reduction in total protein signal compared to fixation with 4% PFA.

Interestingly, assessment of Rab5, which is localized to early endosomes like SNX12 but irreversibly anchored to membranes *via* geranylgeranylation, revealed no difference in total protein detection between 1% and 4% PFA fixation (Figure 4D). However, protein detection in non-permeabilized cells was markedly reduced with 1% PFA fixation. Like transmembrane receptors, Rab5 appears to be properly immobilized with 1% PFA, in contrast to other intracellular proteins we tested. This likely reflects the stable membrane anchorage of Rab5 provided by its lipid modification. Furthermore, Rab5 forms large protein complexes with multiple effectors, including EEA1 and Rabaptin-5, which likely enhance crosslinking efficiency during fixation and thereby improve protein immobilization. In this context, reducing the PFA concentration to 1% clearly improved the accuracy of Rab5 detection in non-permeabilized cells.

Altogether, these results indicate that protein immobilization largely depends on protein type. Efficient immobilization appears to rely on several factors, including strong membrane anchorage, as observed for transmembrane receptors and lipid-anchored proteins, and the presence of interacting protein partners, which may enhance covalent crosslinking and thereby improve protein immobilization.

Finally, we further confirmed the ELISA results with Western blot analyses. Total expression of membrane receptors was similar after fixation with either 1% or 4% PFA, both for receptors predominantly localized at the plasma membrane, such as Myc-AT1-R and Myc-P2Y_12_-R (Supplementary Figure S6A-B), and for receptors exhibiting primarily intracellular localization, such as Myc-V2R W164S, an intracellularly retained mutant of vasopressin V2 receptor (Supplementary Figure S6C). In contrast, total expression of Myc-SNX12, Myc-SNX16 and GAPDH was markedly reduced in cells fixed with 1% PFA compared with 4% PFA (Supplementary Figure S6D-F). These data confirm that 1% PFA fixation can lead to the loss of certain intracellular proteins due to inefficient immobilization. Notably, as these experiments were performed on fixed cells, all proteins of interest may have undergone crosslinking with other proteins, potentially resulting in detection at molecular weights differing from their native forms as a consequence of PFA-induced protein oligomerization.

### Decreasing paraformaldehyde concentration improves the detection accuracy of intracellularly-retained GPCRs

**FIGURE 5:**
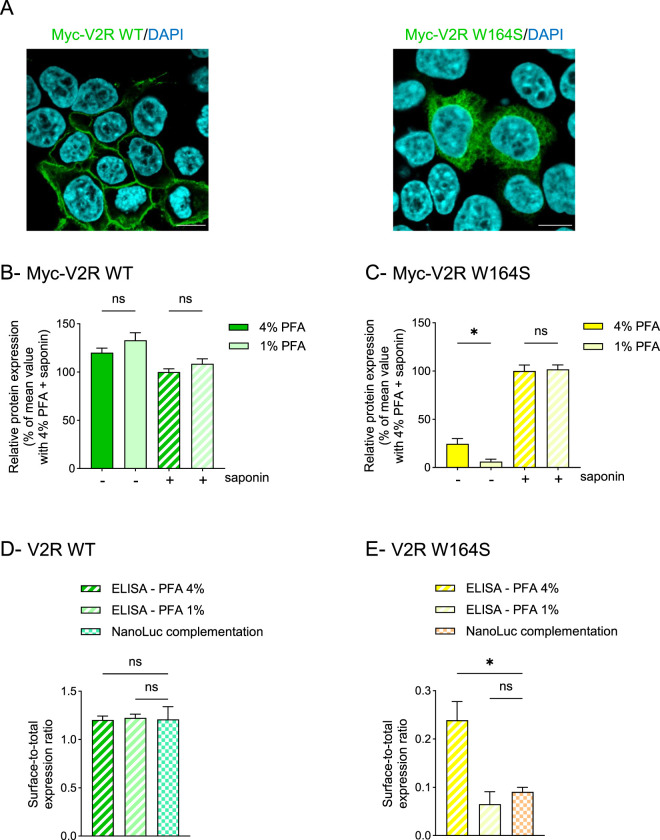
Decreasing paraformaldehyde concentration improves the accurate detection of intracellularly-retained mutant GPCR **(A)**. HEK293T cells were transfected using a lipid-based transfection reagent with vectors encoding Myc-V2R WT or Myc-V2R W164S and then processed for immunofluorescence. Scale bar: 10 μm **(B,C)**. HEK293T cells were transfected using a lipid-based transfection reagent with vectors encoding Myc-V2R WT **(B)** or Myc-V2R W164S **(C)**. Then, cells were fixed using 4% or 1% PFA, permeabilized (+) or not (−) with saponin and receptor expression was quantified by whole-cell ELISA. Data represent the mean ± s.e.m. of four **(B)** or five **(C)** independent experiments. Statistical significance was assessed using one-way ANOVA followed by Sidak’s post-tests (*p < 0.05; ns, not significant). **(D,E)** HEK293T cells were transfected using a lipid-based transfection reagent with vectors encoding HiBiT-V2R WT **(D)** or HiBiT-V2R W164S **(E)** and then processed for NanoLuc complementation to quantify surface and total protein expression. Surface-to-total expression ratios were calculated and compared to those obtained by whole-cell ELISA with 4% or 1% PFA fixation (data from B and C). Data represent the mean ± s.e.m. of four independent experiments. Statistical significance was assessed using one-way ANOVA followed by Sidak’s post-tests (*p < 0.05; ns, not significant).

Developing a robust ELISA protocol for non-permeabilized cells to specifically detect plasma membrane proteins represents a significant challenge in GPCR field, as certain mutations can greatly impact receptor transport to the cell surface. In such cases, it is especially important to accurately quantify plasma membrane expression of the mutant receptor. Therefore, maintaining the integrity of the cell surface and preventing membrane permeabilization is essential to avoid detecting intracellularly-retained receptors. To investigate this point, we focused on the W164S mutation of the vasopressin V2 receptor (V2R), which causes X-linked recessive nephrogenic diabetes insipidus (NDI) and leads to intracellular retention of the receptor, resulting in no or hardly detectable specific agonist binding (Oksche et al., 1996; Morello et al., 2000). In agreement, we observed that Myc-tagged V2R wild-type (WT) was mainly expressed at the plasma membrane of HEK293T cells while Myc-tagged V2R W164S was predominantly localized intracellularly (Figure 5A). In cells expressing Myc-V2R WT, the receptor was quantified similarly in both non-permeabilized and permeabilized cells (Figure 5B), whether the cells were fixed with 4% or 1% PFA, much like Myc-AT1-R (Figure 2A). Conversely, in cells expressing Myc-V2R W164S, the receptor was detected at four-fold lower levels in non-permeabilized cells fixed with 1% PFA than with 4% PFA (Figure 5C, solid bars), while total receptor expression was assessed identically in permeabilized cells, regardless of fixative concentration (Figure 5C, hatched bars). Collectively, these results suggest that membrane permeabilization and the subsequent detection of the intracellular V2R W164S mutant receptor were greatly diminished in cells fixed with 1% PFA while total receptor expression was correctly determined. While about 25% of the mutant receptors were detected in non-permeabilized cells fixed with 4% PFA, decreasing PFA concentration to 1% led to the detection of only 6% of the mutant receptor in non-permeabilized cells.

To confirm that ELISA with 1% PFA fixation provides a more accurate quantification of surface *versus* intracellular receptors than ELISA with 4% PFA, we used the NanoLuc complementation assay, an independent, fixation-free approach that avoids fixation-induced artifacts. In this assay, WT and mutant (W164S) V2R were N-terminally fused to a HiBiT tag, and surface *versus* total receptor expression was quantified by complementation with the Large BiT (LgBiT) protein using non-lytic or lytic buffers, respectively. For V2R WT, which is predominantly localized at the plasma membrane, surface-to-total expression ratios were comparable between ELISA with 4% PFA, ELISA with 1% PFA, and NanoLuc complementation (Figure 5D). Conversely, for the intracellularly retained V2R W164S mutant, the surface-to-total expression ratios measured by NanoLuc complementation were more consistent with that obtained using 1% PFA fixation than with 4% PFA (Figure 5E), indicating that 1% PFA provides a more accurate representation of receptor distribution.

In conclusion, fixation with 1% PFA, rather than 4% PFA, improves the assessment of GPCRs at the cell surface by reducing membrane permeabilization while preserving total receptor content.

## Discussion

The present study reveals critical insights into the effects of paraformaldehyde (PFA) fixation on cell membrane permeability and its implications for accurately quantifying cell surface receptor expression. Our findings indicate that the commonly used 4% PFA concentration induces significant membrane permeabilization, which results in unintended detection of intracellular proteins in non-permeabilized cells.

This phenomenon has profound implications not only for G protein-coupled receptor (GPCR) studies but also for a wide array of biological research techniques that rely on cell surface receptor quantification. These findings are particularly relevant in the context of genetic disorders, where mutations often lead to intracellular retention of misfolded receptors (Ulloa-Aguirre and Conn, 2009; Hegde et al., 2017). Our analysis demonstrated that lower PFA concentrations significantly reduced membrane permeabilization, thereby improving the detection of intracellularly-retained mutant receptors, such as the V2R W164S mutant, thus enabling a more precise measurement of cell surface receptor levels. Accurate assessment of cell surface GPCR expression is not only essential for basic research but also for therapeutic development, particularly in evaluating pharmacological chaperones designed to rescue receptor trafficking and surface expression (Bernier et al., 2004; René et al., 2021). Without reliable quantification, the efficacy of such treatments may be misjudged, underscoring the importance of fixation conditions that preserve membrane integrity while allowing accurate surface receptor detection.

Moreover, the implications of our findings extend beyond the field of GPCR research, impacting a wide range of biological and biomedical studies that rely on PFA fixation. Paraformaldehyde is widely used across various disciplines for its ability to preserve cellular and tissue morphology. However, our study demonstrates that even commonly used fixation protocols can introduce artifacts, particularly by compromising membrane integrity, and highlights the need for critical evaluation of fixation techniques to ensure accurate data interpretation. Indeed, PFA-induced membrane permeabilization could compromise the integrity of data obtained from techniques such as immunocytochemistry, flow cytometry, and enzyme-linked immunosorbent assays (ELISA).

However, identifying suitable chemical alternatives to PFA fixation for surface-restricted immunoassays remains challenging. Alcohol-based fixatives such as methanol or acetone were not considered appropriate for this application, as they are well documented to induce extensive membrane permeabilization through lipid extraction (Jamur and Oliver, 2010). This loss of membrane integrity abolishes the distinction between surface-localized and intracellular protein pools, thereby precluding meaningful interpretation in surface ELISA-based assays. Consequently, their intrinsic permeabilizing properties are fundamentally incompatible with experimental strategies aimed at preserving plasma membrane compartmentalization.

Glutaraldehyde represents another potential fixative due to its strong crosslinking capacity and enhanced protein retention. When applied, glutaraldehyde is typically restricted to very low concentrations (≤0.1%) and used in combination with 4% PFA, as higher concentrations are known to cause extensive epitope masking and severely impair antibody accessibility. These limitations substantially compromise immunodetection sensitivity and specificity, particularly for conformational or extracellular epitopes. For this reason, PFA remains the preferred fixative for immunodetection in surface-specific assays.

Over the last few years, the development of alternative strategies has gained interest due to their ability to assess surface protein expression directly in live cells, thereby avoiding the potential artifacts introduced by fixatives. Innovative techniques such as NanoLuc complementation have emerged as reliable and effective approaches, enabling more accurate and physiologically relevant quantification of both surface and total protein expression (Okashah et al., 2020; Kawakami et al., 2022).

In conclusion, our study highlights the importance of fine-tuning fixation conditions to enhance the accuracy of receptor quantification in biological research. Fixation with 1% PFA offers a viable alternative for studies focused on transmembrane receptors, improving specificity without compromising overall receptor content. For future experiments, it is imperative to systematically tailor protocols based on specific experimental requirements and the type of protein of interest.

## Materials and methods

### Expression vectors

Rat AT1-R was fused to an HA tag (Saulière et al., 2012) or a Myc tag at the extracellular N-terminus. SNX12, SNX16, P2Y_12_-R and α2c-AR were Myc-tagged as previously described (Brankatschk et al., 2011; Pons et al., 2012; 2014; Bellot et al., 2015), and SNX12 was also fused to an HA tag at the N-terminus. Myc-tagged V2R WT and W164S were obtained from Michel Bouvier (Canada) and were also fused to HiBiT tag at the N-terminus. PDGFRα was fused to a Myc tag at the intracellular C-terminus, TfR to an HA tag at the extracellular C-terminus region and Rab5 to a Myc tag.

### Cell culture and transfection

HEK293T/17 cells (human embryonic kidney) or HeLa cells were maintained in DMEM AQmedia (Sigma. St. Louis, MO) supplemented with 10% fetal bovine serum (Life technologies) and 100 units/mL penicillin and 100 μg/mL streptomycin at 37 °C in a humidified atmosphere containing 5% CO_2_. Twenty-four hours after splitting, cells were transiently transfected using either the lipid-based X-tremeGENE^TM^ 9 DNA Transfection Reagent (Sigma-Aldrich/Merck, Darmstadt, Germany) at a 3:1 reagent-to-DNA ratio or the synthetic cationic polymer polyethylenimine (PEI, Polysciences Inc.) at a 2:1 reagent-to-DNA ratio. For all conditions, the total plasmid DNA amount was kept constant; the plasmid encoding the protein of interest was varied as needed, and the remaining DNA was supplemented with empty vector to maintain the total DNA quantity.

### Microscopy

Cells were seeded onto poly-D-lysine-coated glass coverslips in a 6-well plate, cultured for 24 h and then transfected with 1 μg plasmid DNA and 3 μL X-tremeGENE^TM^ 9 DNA Transfection Reagent. Forty-eight hours after transfection, cells were fixed with 4% paraformaldehyde, saturated with PBS containing 1% bovine serum albumin (BSA) and incubated with the primary antibody (anti-Myc (Clone 9E10.1:100. Santa Cruz Biotechnology. Dallas, Texas, United States); anti-HA (Clone 16B12.1:100. BioLegend. San Diego. California, United States); anti-vinculin (Clone 7F9. 1:100. Santa Cruz Biotechnology. Dallas, Texas, United States); anti-GAPDH antibody (Clone D16H11.1:100. Cell Signaling. Danvers, Massachusetts, United States) and then with Alexa Fluor® 488-conjugated fluorescent secondary antibody (Life technologies), both in the presence of 0.05% saponin to achieve membrane permeabilization. Nuclei were stained with 5 μg/mL DAPI (,6-diamidino-2-phenylindole) for 30 min. Confocal images were captured with a LSM 780 operated with Zen software using a 63x, 1.4 NA Plan-Apochromat objective (Carl Zeiss).

### Quantification of protein expression by whole-cell ELISA

Relative protein expression was quantified as previously described (Garcia et al., 2019). Briefly, HEK293T/17 or HeLa cells were split into 24-well plates pre-coated with Poly-D-lysine, transiently transfected with a control empty vector (pcDNA3.1) or vector encoding N-terminally Myc- or HA-tagged protein of interest.

To ensure accurate quantification, preliminary transfection experiments were systematically performed using increasing amounts of plasmid encoding the proteins of interest (from 12.5 to 250 ng per well). Protein expression was quantified by ELISA to identify DNA concentrations yielding signals within the linear dynamic range of the assay and to avoid saturation. All quantitative analyses reported in this study were subsequently performed using plasmid amounts validated to fall within this range.

For HEK293T/17 cells transfected with X-tremeGENE^TM^ 9 DNA Transfection Reagent, the plasmid DNA amounts per well of a 24-well plate were as follows: 12.5 ng for Myc-V2R WT; 25 ng for Myc-AT1-R, Myc-SNX12, HA-AT1-R, HA-SNX12, Myc-P2Y_12_-R, PDGFRα-Myc, Myc-SNX16; 250 ng for Myc-α2c-AR, TfR-HA, Myc-Rab5, Myc-V2R W164S. For HEK293T/17 cells transfected with PEI or for HeLa cells transfected with X-tremeGENE^TM^ 9, a plasmid DNA amount of 250 ng per well was used. For all transfections, the amounts of plasmids encoding the proteins of interest were always supplemented with empty vector to maintain a constant total DNA amount across conditions.

Forty-eight hours post-transfection, cells were first washed then fixed with 4% or 1% paraformaldehyde (for the indicated time period), saturated (PBS - 1% BSA) for 45 min and incubated for 1 h with the primary antibody (anti-Myc (Clone 9E10.1:500. Santa Cruz Biotechnology. Dallas, Texas, United States); anti-HA (Clone 16B12.1:1000. BioLegend. San Diego. California, United States); anti-vinculin (Clone 7F9. 1:500. Santa Cruz Biotechnology. Dallas, Texas, United States); anti-GAPDH antibody (Clone D16H11.1:500. Cell Signaling. Danvers, Massachusetts, United States). After two washes, cells were incubated for 1 h with horseradish peroxidase-labeled secondary antibody (Sigma-Aldrich/Merck. 1:1000. Darmstadt, Germany). After two additional washes, cells were incubated with 500 µL HRP substrate: TMB (3,39,5,59-tetramethylbenzidine) (Becton Dickinson, New Jersey, United States). The reaction was stopped with 125 µL HCl 1N. 200 µL of reaction mixture were transferred into a 96-well microplate for reading at 450 nm in a microplate reader (Infinite F500. Tecan Group Ltd. Männedorf, Switzerland). The background value obtained from control cells transfected with an empty vector (pcDNA3.1) was subtracted from all values obtained from cells expressing the protein of interest. When specified, cell membrane permeabilization was achieved by adding 0.05% saponin into the antibody solutions.

### Quantification of protein expression by NanoLuc complementation

Relative protein expression was quantified using the Nano-Glo® HiBiT Detection System (Promega Corporation). HEK293T/17 were seeded in 6-well plates and transiently transfected with either a control empty vector (pcDNA3.1) or plasmid encoding N-terminally HiBiT-tagged receptor of interest. Forty-eight hours after transfection, cells were washed, resuspended in PBS containing 0.1% (w/v) glucose at room temperature and distributed in a white 96-well microplate (PerkinElmer). Plasma membrane and total levels of HiBiT-tagged receptors were quantified using non-lytic or lytic detection reagents, respectively, both containing Large BiT protein (LgBiT) and the furimazine NanoLuc substrate. Binding of LgBiT to HiBiT-tagged receptors reconstitutes an active NanoLuc luciferase through structural complementation, enabling quantitative detection of luminescence signals. Luminescence was measured 5 min after substrate addition using a modified Infinite F500 (Tecan Group Ltd.). Background luminescence from cells transfected with the empty vector was subtracted from all measurements.

#### Western blotting

To mimic the different steps of the ELISA protocol, HEK293T/17 cells were similarly split into Poly-D-lysine-pre-coated 6-well plates, transiently transfected with a control empty vector (pcDNA3.1) or vector encoding N-terminally Myc-tagged protein of interest. Forty-eight hours post-transfection, cells were first washed then fixed with 4% or 1% paraformaldehyde for 10 min, saturated (PBS - 1% BSA) for 45 min and incubated with PBS-BSA 1% containing 0.05% saponin for 1 h to mimic the incubation with the primary antibody. Then cells were washed and again incubated with PBS-BSA 1% containing 0.05% saponin for 1 h to mimic the incubation with the secondary antibody. After washing, cells were directly scraped in a lysis buffer (25 mM Tris-HCl pH 7.4, 140 mM NaCl, 2 mM EDTA, 0.5% n-dodecyl-β-D-maltoside (DDM)) in the presence of a protease inhibitor cocktail (Roche). The protein concentration of extracts was determined by the Lowry method (Bio-Rad) and equal amounts of proteins (25 µg) were subjected to SDS-PAGE and transferred to nitrocellulose membranes (Millipore). Proteins were detected with primary (anti-Myc antibody (Clone 9E10.1:1000. Santa Cruz Biotechnology. Dallas, Texas, United States); anti-GAPDH antibody (Clone D16H11.1:1000. Cell Signaling. Danvers, Massachusetts, United States)) followed by horseradish peroxidase-conjugated secondary antibodies (GE Healthcare) using an enhanced chemiluminescence detection reagent (GE Healthcare). Protein quantification was obtained by densitometric analysis using ImageJ software.

## Data Availability

The raw data supporting the conclusions of this article will be made available by the authors, without undue reservation.
