# Supplementary material for: Assessing the limitations of paraformaldehyde fixation for accurate cell surface receptor measurement
Source: Front Pharmacol. 2026 Mar 24;17:1727410. doi: 10.3389/fphar.2026.1727410 (PMC13055513; doi:10.3389/fphar.2026.1727410)
Supplement: Supplementary file 1 [file Supplementaryfile1.pptx]

## Slide 1
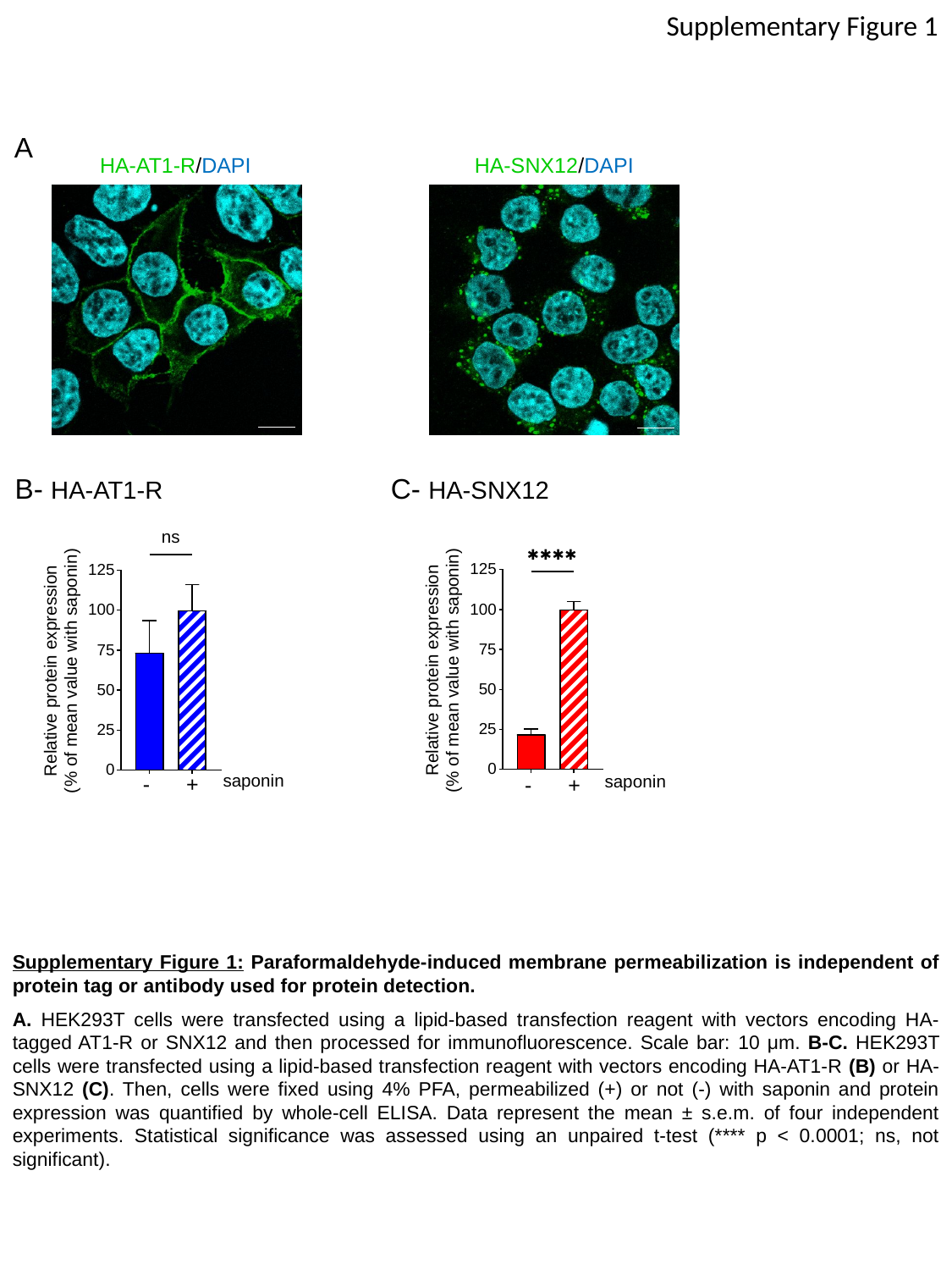

Supplementary Figure 1
A
HA-AT1-R/DAPI
HA-SNX12/DAPI
B- HA-AT1-R
C- HA-SNX12
Supplementary Figure 1: Paraformaldehyde-induced membrane permeabilization is independent of protein tag or antibody used for protein detection.
A. HEK293T cells were transfected using a lipid-based transfection reagent with vectors encoding HA-tagged AT1-R or SNX12 and then processed for immunofluorescence. Scale bar: 10 μm. B-C. HEK293T cells were transfected using a lipid-based transfection reagent with vectors encoding HA-AT1-R (B) or HA-SNX12 (C). Then, cells were fixed using 4% PFA, permeabilized (+) or not (-) with saponin and protein expression was quantified by whole-cell ELISA. Data represent the mean ± s.e.m. of four independent experiments. Statistical significance was assessed using an unpaired t-test (**** p < 0.0001; ns, not significant).

## Slide 2
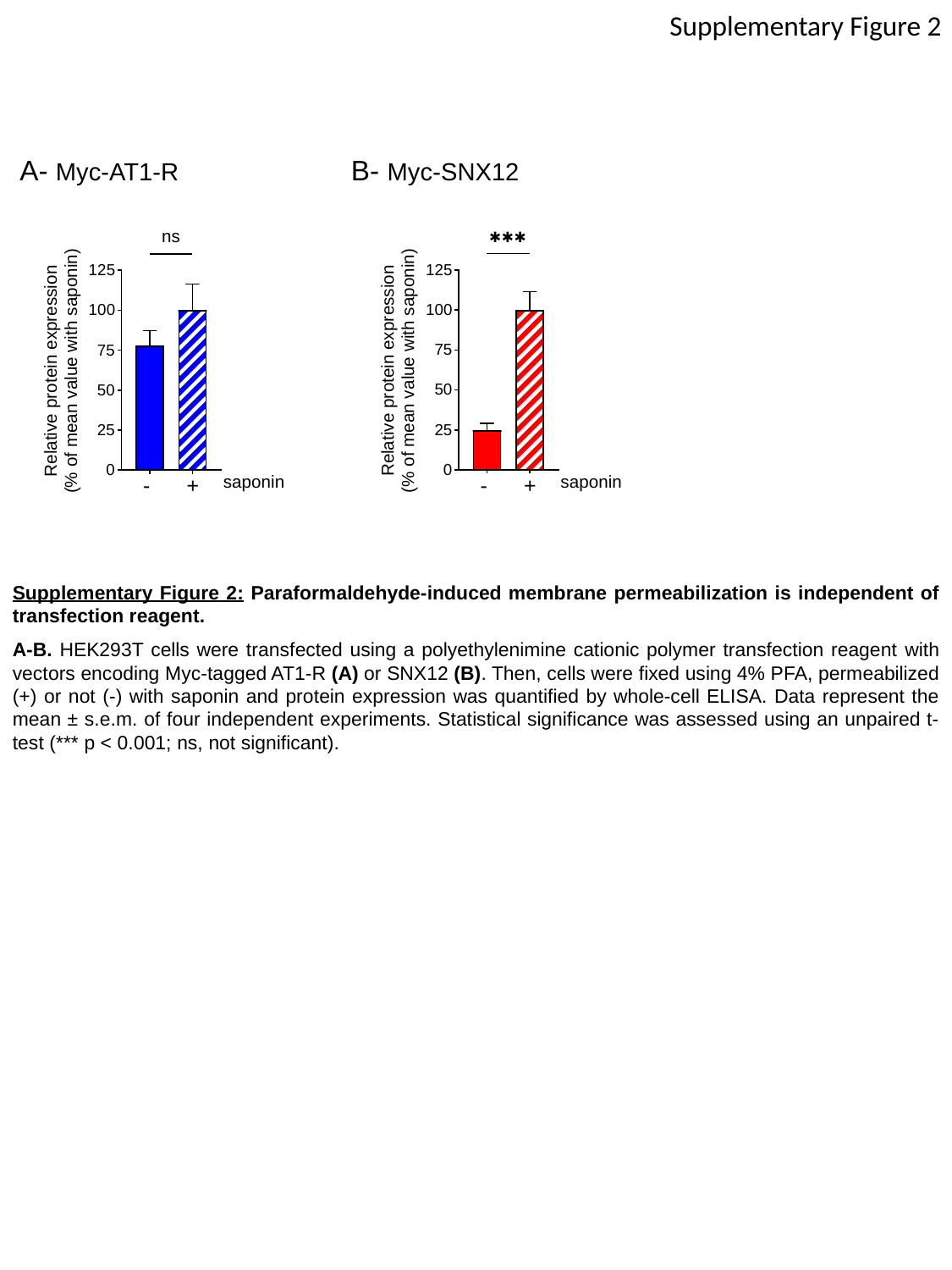

Supplementary Figure 2
A- Myc-AT1-R
B- Myc-SNX12
Supplementary Figure 2: Paraformaldehyde-induced membrane permeabilization is independent of transfection reagent.
A-B. HEK293T cells were transfected using a polyethylenimine cationic polymer transfection reagent with vectors encoding Myc-tagged AT1-R (A) or SNX12 (B). Then, cells were fixed using 4% PFA, permeabilized (+) or not (-) with saponin and protein expression was quantified by whole-cell ELISA. Data represent the mean ± s.e.m. of four independent experiments. Statistical significance was assessed using an unpaired t-test (*** p < 0.001; ns, not significant).

## Slide 3
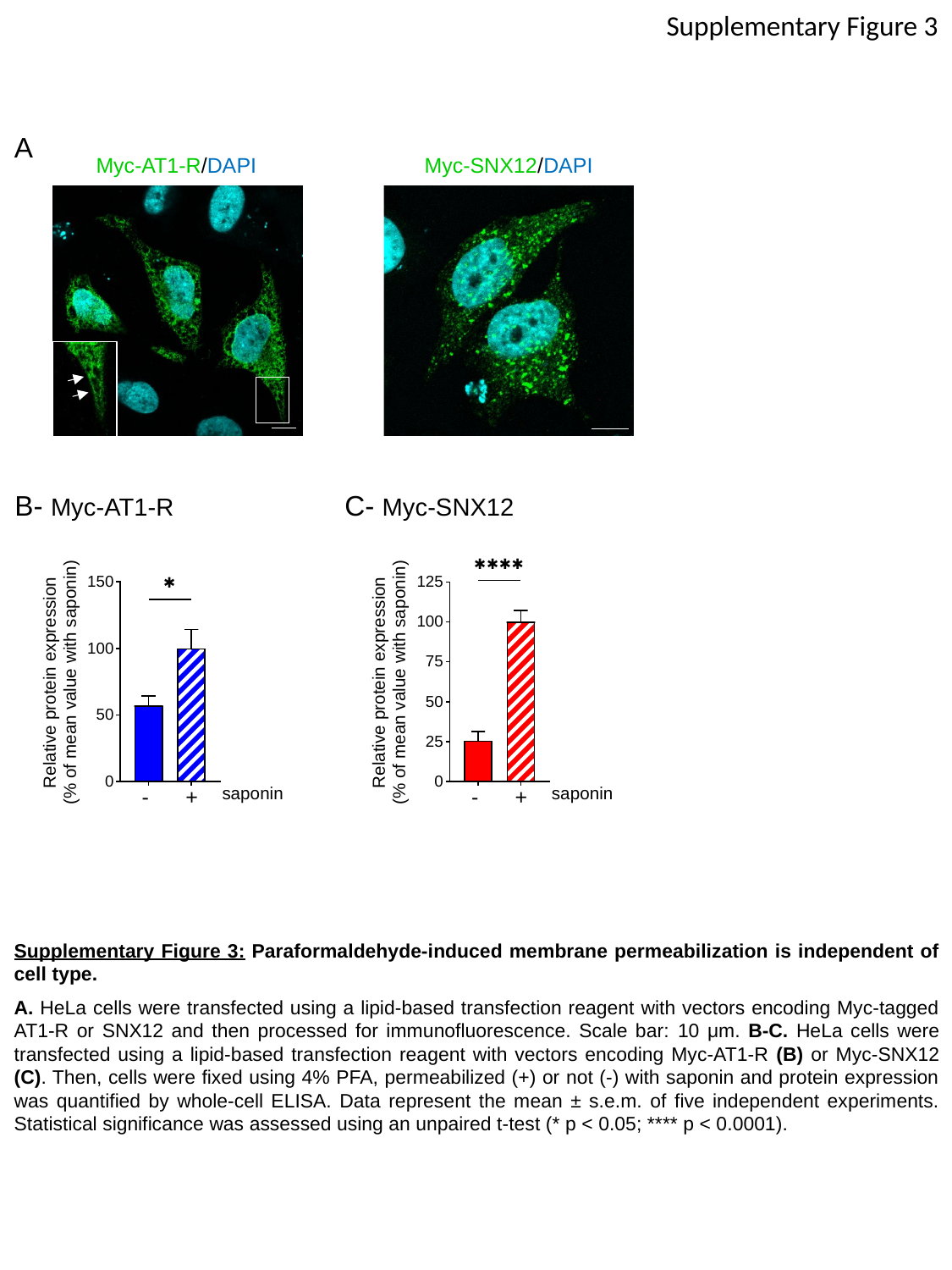

Supplementary Figure 3
A
Myc-AT1-R/DAPI
Myc-SNX12/DAPI
B- Myc-AT1-R
C- Myc-SNX12
Supplementary Figure 3: Paraformaldehyde-induced membrane permeabilization is independent of cell type.
A. HeLa cells were transfected using a lipid-based transfection reagent with vectors encoding Myc-tagged AT1-R or SNX12 and then processed for immunofluorescence. Scale bar: 10 μm. B-C. HeLa cells were transfected using a lipid-based transfection reagent with vectors encoding Myc-AT1-R (B) or Myc-SNX12 (C). Then, cells were fixed using 4% PFA, permeabilized (+) or not (-) with saponin and protein expression was quantified by whole-cell ELISA. Data represent the mean ± s.e.m. of five independent experiments. Statistical significance was assessed using an unpaired t-test (* p < 0.05; **** p < 0.0001).

## Slide 4
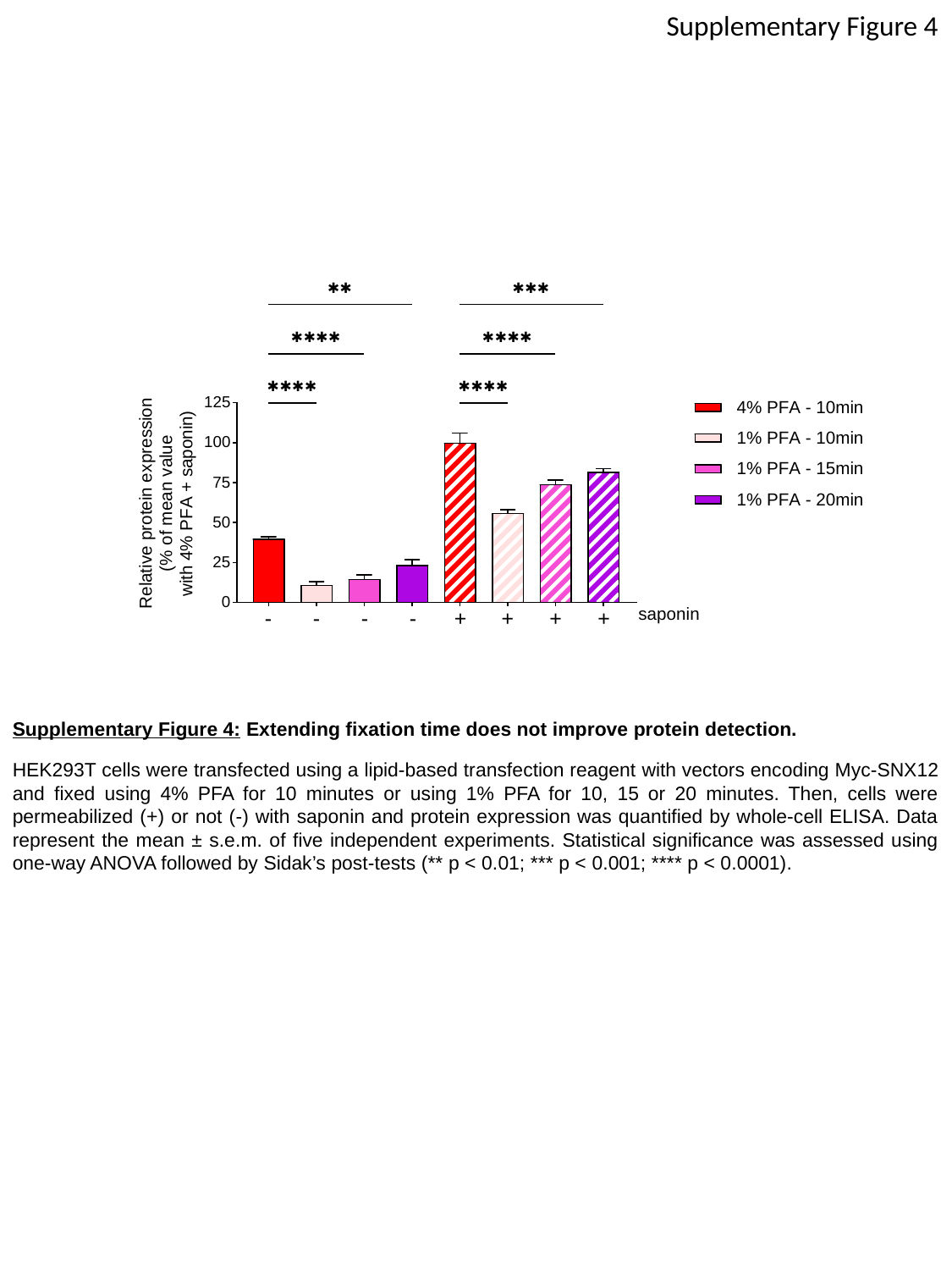

Supplementary Figure 4
Supplementary Figure 4: Extending fixation time does not improve protein detection.
HEK293T cells were transfected using a lipid-based transfection reagent with vectors encoding Myc-SNX12 and fixed using 4% PFA for 10 minutes or using 1% PFA for 10, 15 or 20 minutes. Then, cells were permeabilized (+) or not (-) with saponin and protein expression was quantified by whole-cell ELISA. Data represent the mean ± s.e.m. of five independent experiments. Statistical significance was assessed using one-way ANOVA followed by Sidak’s post-tests (** p < 0.01; *** p < 0.001; **** p < 0.0001).

## Slide 5
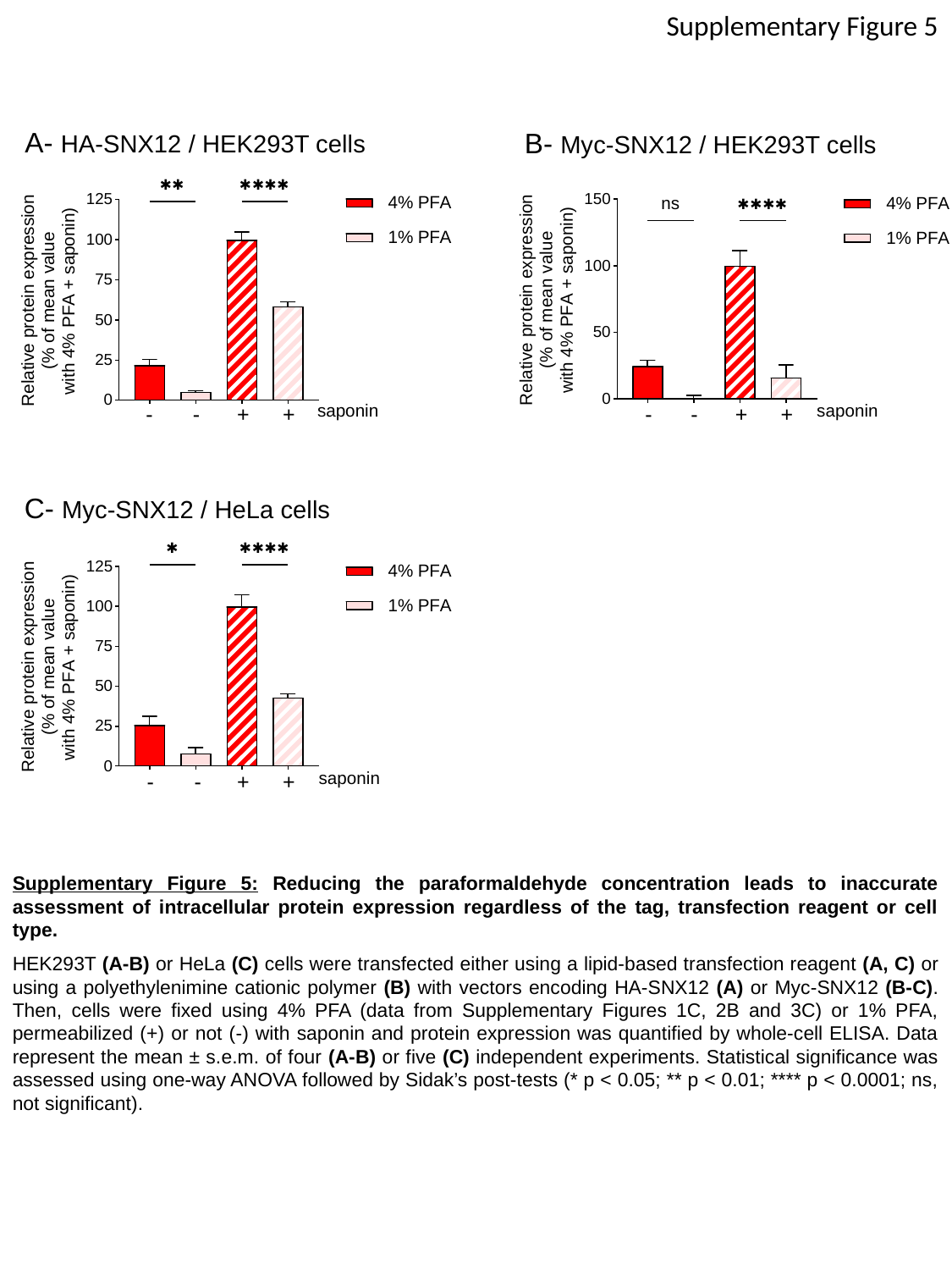

Supplementary Figure 5
A- HA-SNX12 / HEK293T cells
B- Myc-SNX12 / HEK293T cells
C- Myc-SNX12 / HeLa cells
Supplementary Figure 5: Reducing the paraformaldehyde concentration leads to inaccurate assessment of intracellular protein expression regardless of the tag, transfection reagent or cell type.
HEK293T (A-B) or HeLa (C) cells were transfected either using a lipid-based transfection reagent (A, C) or using a polyethylenimine cationic polymer (B) with vectors encoding HA-SNX12 (A) or Myc-SNX12 (B-C). Then, cells were fixed using 4% PFA (data from Supplementary Figures 1C, 2B and 3C) or 1% PFA, permeabilized (+) or not (-) with saponin and protein expression was quantified by whole-cell ELISA. Data represent the mean ± s.e.m. of four (A-B) or five (C) independent experiments. Statistical significance was assessed using one-way ANOVA followed by Sidak’s post-tests (* p < 0.05; ** p < 0.01; **** p < 0.0001; ns, not significant).

## Slide 6
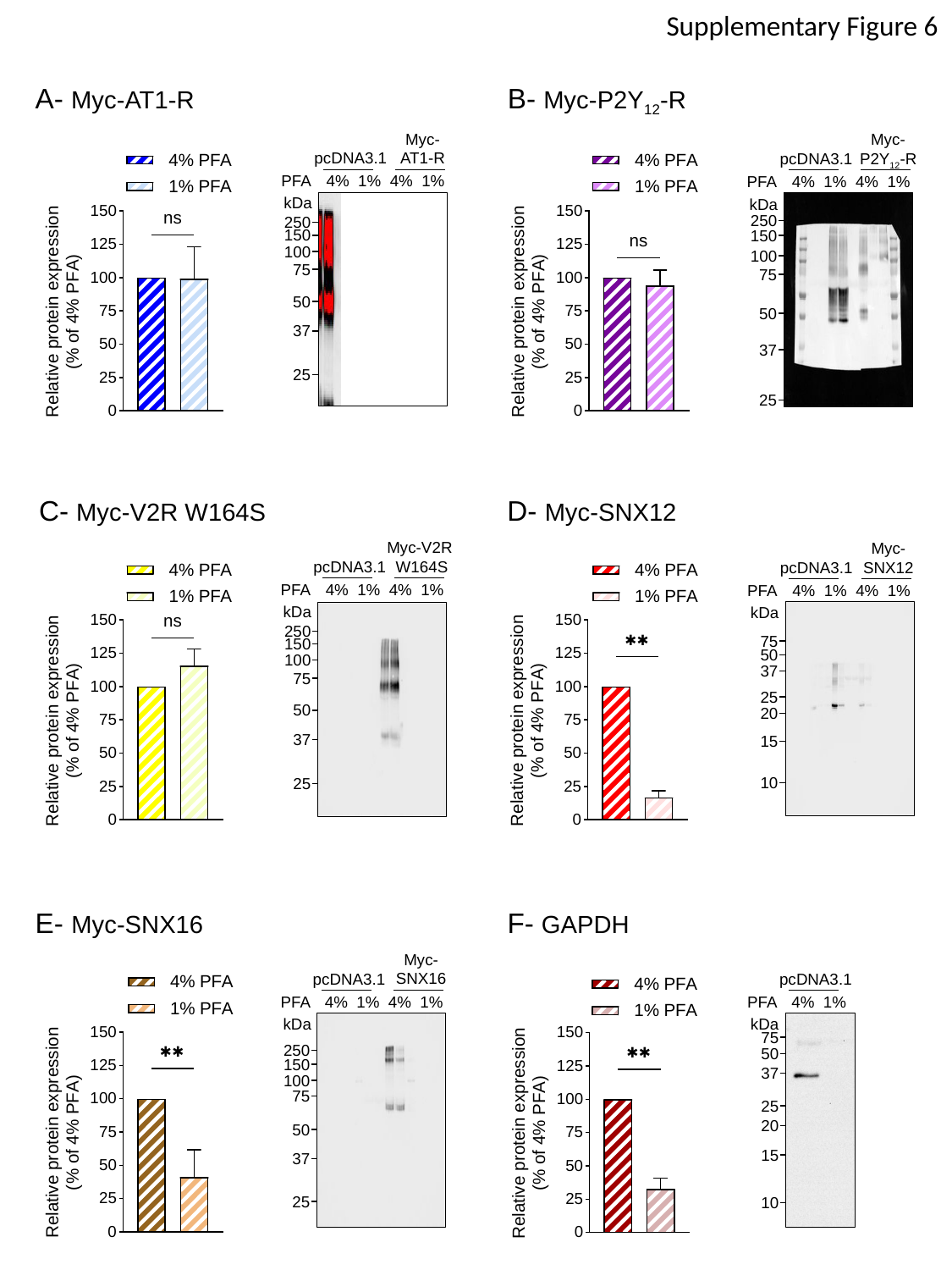

Supplementary Figure 6
A- Myc-AT1-R
B- Myc-P2Y12-R
Myc-
AT1-R
pcDNA3.1
PFA
4%
1%
4%
1%
kDa
Myc-
P2Y12-R
pcDNA3.1
PFA
4%
1%
4%
1%
kDa
250
150
100
75
50
37
25
250
150
100
75
50
37
25
C- Myc-V2R W164S
D- Myc-SNX12
Myc-V2R
W164S
pcDNA3.1
PFA
4%
1%
4%
1%
kDa
250
150
100
75
50
37
25
Myc-
SNX12
pcDNA3.1
PFA
4%
1%
4%
1%
kDa
75
50
37
25
20
15
10
E- Myc-SNX16
F- GAPDH
Myc-
SNX16
pcDNA3.1
4%
1%
PFA
pcDNA3.1
4%
1%
PFA
kDa
75
50
37
25
20
15
10
4%
1%
kDa
250
150
100
75
50
37
25

## Slide 7
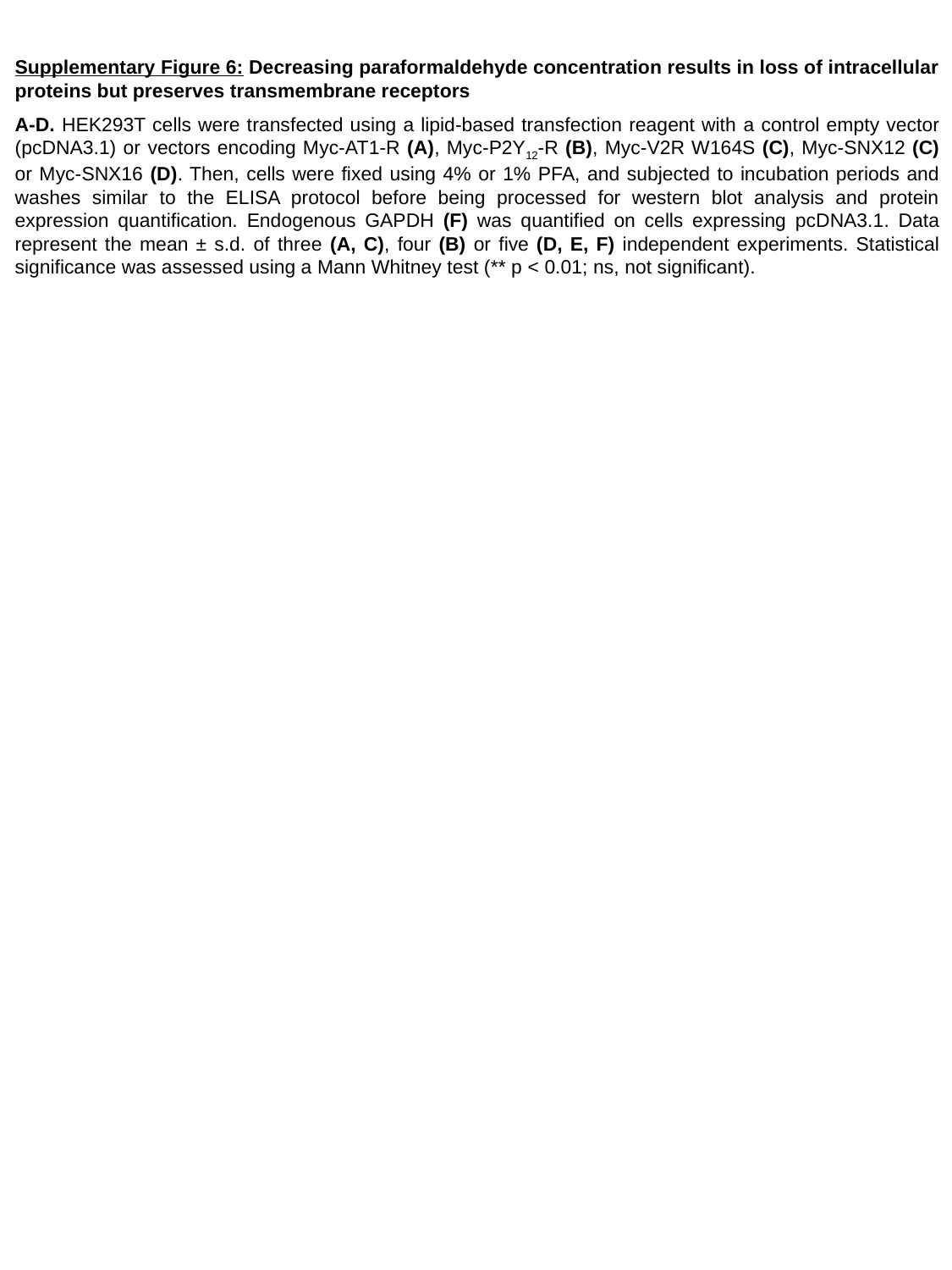

Supplementary Figure 6: Decreasing paraformaldehyde concentration results in loss of intracellular proteins but preserves transmembrane receptors
A-D. HEK293T cells were transfected using a lipid-based transfection reagent with a control empty vector (pcDNA3.1) or vectors encoding Myc-AT1-R (A), Myc-P2Y12-R (B), Myc-V2R W164S (C), Myc-SNX12 (C) or Myc-SNX16 (D). Then, cells were fixed using 4% or 1% PFA, and subjected to incubation periods and washes similar to the ELISA protocol before being processed for western blot analysis and protein expression quantification. Endogenous GAPDH (F) was quantified on cells expressing pcDNA3.1. Data represent the mean ± s.d. of three (A, C), four (B) or five (D, E, F) independent experiments. Statistical significance was assessed using a Mann Whitney test (** p < 0.01; ns, not significant).
